# Employing individual measures of baseline glucocorticoids as population-level conservation biomarkers: considering within-individual variation in a breeding passerine

**DOI:** 10.1093/conphys/cow048

**Published:** 2016-10-15

**Authors:** Christine L Madliger, Oliver P Love

**Affiliations:** 1 Department of Biological Sciences, University of Windsor, 401 Sunset Avenue, Windsor, Ontario, Canada N9B 3P4; 2 Great Lakes Institute for Environmental Research, University of Windsor, 401 Sunset Avenue, Windsor, Ontario, Canada N9B 3P4

**Keywords:** Biomarker, consistency, corticosterone, individual variation, repeatability, tree swallow

## Abstract

Labile physiological variables, such as stress hormones [i.e. glucocorticoids (GCs)], allow individuals to react to perturbations in their environment and may therefore reflect the effect of disturbances or positive conservation initiatives in advance of population-level demographic measures. Although the application of GCs as conservation biomarkers has been of extensive interest, few studies have explicitly investigated whether baseline GC concentrations respond to disturbances consistently across individuals. However, confirmation of consistent responses is of paramount importance to assessing the ease of use of GCs in natural systems and to making valid interpretations regarding population-level change (or lack of change) in GC concentrations. We investigated whether free-ranging female tree swallows (*Tachycineta bicolor*) display individually specific changes in baseline glucocorticoid concentrations naturally over the breeding season (from incubation to offspring provisioning) and in response to a manipulation of foraging profitability (representing a decrease in access to food resources). We show that baseline GC concentrations are repeatable within individuals over reproduction in natural conditions. However, in response to a reduction in foraging ability, baseline GC concentrations increase at the population level but are not repeatable within individuals, indicating a high level of within-individual variation. Overall, we suggest that baseline GCs measured on a subset of individuals may not provide a representative indication of responses to environmental change at the population level, and multiple within-individual measures may be necessary to determine the fitness correlates of GC concentrations. Further validation should be completed across a variety of taxa and life-history stages. Moving beyond a traditional cross-sectional approach by incorporating repeated-measures methods will be necessary to assess the suitability of baseline GCs as biomarkers of environmental change and population persistence, particularly from a logistical and ease-of-use perspective for conservation managers.

## Teaser

We outline the importance of considering within-individual changes in physiology when determining how disturbances may impact wildlife. Our results indicate that stress hormones measured on a subset of individuals may not provide a clear picture of how entire populations could respond to environmental change; multiple measures from the same individuals over time may be necessary.

## Introduction

### Glucocorticoids as conservation biomarkers

Conservation biologists require a diverse toolbox to identify, ameliorate and predict threats to wildlife and to monitor the outcome of management initiatives (Bradshaw and Brook, 2010). The growing discipline of conservation physiology focuses on documenting how organisms respond to changes in their environment and, potentially, offers a unique set of predictive tools (Wikelski and Cooke, 2006; Cooke *et al*., 2013). In particular, the labile physiological processes related to metabolism, energetics, immune function, reproduction and oxidative status can be sensitive to internal and external environmental factors (Carey, 2005; Stevenson *et al*., 2005; Walker *et al*., 2005; Cooke *et al*., 2013). Although many physiological traits are available as potential biomarkers (for an overview, see Cooke *et al*., 2013), glucocorticoids [GCs; corticosterone (CORT) and cortisol] have been widely used for inferring disturbance across a variety of taxa (Busch and Hayward, 2009; Dantzer *et al*., 2014), largely because of their function in allowing organisms to respond acutely to unexpected perturbations in their environment (McEwen and Wingfield, 2003; Wingfield, 2005; Busch and Hayward, 2009).

Glucocorticoids are metabolic hormones involved in the maintenance of energetic balance through their influences on glucose and lipid metabolism (Landys *et al*., 2006) and are commonly associated with their role in the acute stress response (Sapolsky *et al*., 2000). In the face of an unexpected perturbation in the environment, GC concentrations increase to promote the mobilization of stored energy sources, regulate immune function, promote escape behaviour and suppress non-essential activities, such as reproduction, in the subsequent minutes to hours (Sapolsky *et al*., 2000; Wingfield and Kitaysky, 2002). Glucocorticoids also play a constant and essential role at baseline levels by promoting foraging and metabolism to maintain adequate glucose and fatty acid concentrations, leading to predictable variation over diel (Landys *et al*., 2006) and seasonal cycles (Romero, 2002). Baseline GCs often increase during predictable periods of energetic demand (Wingfield, 2005), such as offspring provisioning (Romero, 2002). Baseline circulating GCs and integrated measures of GCs, such as those found in faeces and outer integuments, have also been shown, in some cases, to respond to changes in environmental quality (Busch and Hayward, 2009; Baker *et al*., 2013) and relate to fitness (reviewed by Bonier *et al*., 2009a), further supporting their potential use as a monitoring tool for rapidly detecting disturbance in wildlife populations.

Although the potential value of baseline GCs for conservation is well established (Wingfield *et al*., 1997; Busch and Hayward, 2009; Baker *et al*., 2013), the contribution of GCs to on-the-ground conservation success has been relatively limited (Madliger *et al*., 2016). Glucocorticoids have been used as monitoring tools (often in combination with other physiological metrics) to infer disturbance and ultimately influence habitat regulations in certain systems, such as killer whales (*Orcinus orca*; Ayres *et al*., 2012), yellow-eyed penguins (*Megadyptes antipodes*; Ellenberg *et al*., 2006, 2007) and woodland caribou (*Rangifer tarandus*; Wasser *et al*., 2011; Joly *et al*., 2015). However, given the attention they have amassed in the conservation physiology research literature (Lennox and Cooke, 2014), implementation has been slower than might be expected. One potential reason for this disconnect may be that GCs have not been fully validated as conservation biomarkers, making it difficult for conservation practitioners to know when they will be effective monitoring tools for specific species or populations.

### Importance of within-individual variation

In particular, a characteristic that has been understudied within the context of applying GCs to conservation is the amount of variation in GC concentrations that occurs between, vs. within, individuals (i.e. repeatability; Killen *et al*., 2016). Validating consistency in GC responses is important, as follows: (i) to ensure that measurements obtained from random subsets of individuals will be representative of population-level processes; and (ii) to determine whether multiple measures of baseline GCs may be necessary to predict performance or fitness (i.e. to properly assess the predictive capacity of baseline GCs; Dantzer *et al*., 2014; Madliger and Love, 2014, 2015). Given recent work indicating that GC responses to prolonged stressors can vary markedly across species, populations, or between captive and wild systems (Dickens and Romero, 2013), the potential for individually specific responses to environmental change within a population warrants explicit consideration.

**Figure 1:**
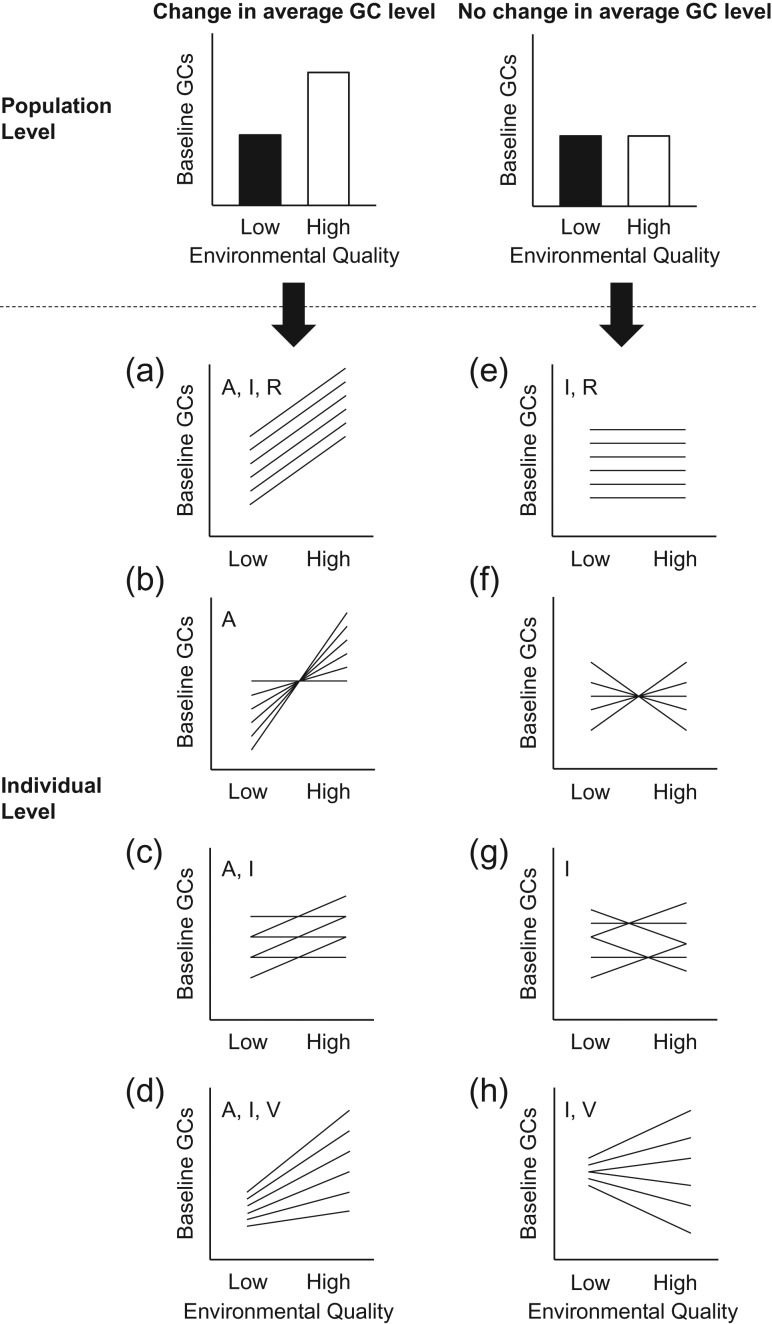
Diagram showing the ways in which within-individual (i.e. repeated-measures) data can underlie patterns at the average (population) level. Each line represents a different individual. The eight patterns can be distinguished based on the following four characteristics: (i) a change in baseline glucocorticoids (GCs) on average (A in **a–d**); (ii) differences in baseline GC concentrations between individuals (I in a, c, d, **e**, **g** and **h**); (iii) significant consistency repeatability within individuals (R in a and e); and (iv) a change in variance of baseline GCs across environments (V in d and h). Figure adapted from Nussey *et al*. (2007).

The most common approach for using GC concentrations to ascertain the influence of a disturbance or change in environmental quality on wildlife has been to compare the average hormone concentrations of populations at sites with differing exposure (e.g. pristine vs. degraded; Fig. 1, upper panel). Drawing conclusions about the population from this type of cross-sectional approach necessarily assumes that all individuals respond (or do not respond) to a given environmental change in a similar way (e.g. that all individuals will display an increase in GC concentrations in response to the habitat alteration; Fig. 1a). However, individuals may react in individually specific ways to a change in environmental quality, and ignoring this inherent possibility can lead to invalid interpretations of GC concentrations at the average (population) level (Williams, 2008; Dingemanse *et al*., 2010). Specifically, an approach that compares only average GC concentrations would conclude that scenarios a, b, c and d in Fig. 1 are equivalent, leading to a number of potential complications for interpreting baseline GC concentrations as indicators of environmental disturbance.

First, if baseline GCs change in individually specific ways (as in Fig. 1b, c and d), the ability to measure subsets of individuals over time and consider them as representative of the population becomes more difficult, and the potential to detect a disturbance sensitively with baseline GC concentrations diminishes (Madliger and Love, 2014), especially when sample sizes are low (as can be the case in conservation situations).

Second, if certain individuals in a population disproportionately influence population persistence and are also disproportionately impacted by a disturbance (Fefferman and Romero, 2013), then measurements at the average (cross-sectional) level may incorrectly predict future population change. Only through a repeated-measures approach is it possible to identify whether individuals with certain phenotypes are the most susceptible to environmental changes (e.g. whether individuals with high GC concentrations before environmental change respond in a different manner from individuals with low initial GC concentrations).

Third, acquiring multiple baseline GC measures from the same individual over time allows consistency (i.e. repeatability) to be assessed, which can determine whether individuals maintain a given ‘baseline GC phenotype’ over time. This provides important information about the time frames over which baseline GC measures can be grouped, considered equivalent and viewed as representative of an individual's physiological status.

Fourth, and following from the previous point, incorporating a repeated-measures approach is required to determine whether single or multiple measures of baseline GCs may be necessary when predicting fitness (i.e. determining the predictive capacity of GCs and therefore their utility for conservation managers; Dingemanse *et al*., 2010). Indeed, there is growing evidence that the individual management of GC concentrations over key time periods may better predict fitness than static measures (Love and Williams, 2008; Bonier *et al*., 2011; Ouyang *et al*., 2011b, 2013; Arlettaz *et al*., 2015; Love *et al*., 2014); individuals that show high within-individual variation in GC physiology may be more (or less) susceptible to environmental disturbance (Killen *et al*., 2016; Taff and Vitousek, 2016).

Finally, limiting investigations to a cross-sectional approach may lead to the conclusion that GC concentrations are stable (Fig. 1e, f, g and h) despite a high level of within-individual variation (Fig. 1f, g and h). However, this within-individual variation could be indicative of a physiological disturbance, signalling important fitness consequences with implications for population health and persistence. Overall, experiments where the same individual is measured in both the control and the altered environment (i.e. a repeated-measures approach) are necessary to reveal whether we can have confidence that a cross-sectional monitoring approach will be informative for the population.

### Calculating within-individual variation

Ecological and evolutionary ecologists have long been interested in quantifying between- and within-individual variation for the purpose of studying behavioural syndromes (Bell, 2007; Dingemanse and Dochtermann, 2013), quantifying the heritability and selective potential of a diversity of traits (Lynch and Walsh, 1998) and determining the fitness consequences of individual flexibility (Piersma and Drent, 2003; Ghalambor *et al*., 2007; Nussey *et al*., 2007). The consistency of traits (physiological or otherwise) is most often ascertained through the calculation of repeatability, which refers to the amount of variation in a trait that is attributable to between- rather than within-individual differences (Lessells and Boag, 1987). Repeatability estimates of baseline GCs have been mixed, differing depending on factors such as season, length of time between measurements, sampling conditions (e.g. wild vs. laboratory settings) and other environmental factors (Ouyang *et al*., 2011a). For example, although high repeatability of baseline GCs has been found previously within the breeding season in tree swallows (*Tachycineta bicolor*) and great tits (*Parus major*; Ouyang *et al*., 2011a), repeatability estimates have generally been low over longer time spans (months to years) in these same species (Ouyang *et al*., 2011a) and in largemouth bass (*Micropterus salmoides*; Cook *et al*., 2011), Florida scrub jays (*Aphelocoma coerulescens*; Rensel and Schoech, 2011) and garter snakes (*Thamnophis elegans*; Sparkman *et al*., 2014). However, this pattern is not without deviation, as Angelier *et al*. (2010) found high repeatability of baseline GC concentrations over a 1 year period in breeding black-browed albatrosses (*Thalassarche melanophris*), and Pavitt *et al*. (2015) found high repeatability of faecal GC metabolites over a 10 year sampling period in wild red deer (*Cervus elaphus*), but only after accounting for age and season. Although the repeatability of baseline GCs has been investigated over these various time spans and seasons in wild settings, and attention has been paid to individual variation in stress-induced responses to standardized restraint protocols (reviewed by Cockrem, 2013), considerably less attention has been paid to whether repeatability of baseline GCs occurs in the context of extended environmental challenge (e.g. Cook *et al*., 2011).

It is integral to note that there are multiple ways to assess repeatability (Nakagawa and Schielzeth, 2010; Biro and Stamps, 2015). ‘Agreement repeatability’ has most traditionally been applied in behavioural and physiological systems to determine whether individuals maintain the same trait value across time (Lessells and Boag, 1987; Nakagawa and Schielzeth, 2010). However, a standardized form of repeatability known as ‘consistency repeatability’ can provide information on whether individuals show consistency in directional responses (i.e. if all individuals change in a similar manner over time; Biro and Stamps, 2015). Therefore, the analytical and statistical tools required to assess within-individual consistency over changing environments are readily available. Indeed, longitudinal (i.e. repeated-measures) data sets are being increasingly incorporated in ecological physiology, providing an opportunity to transfer analytical approaches to conservation physiology applications as well.

### Case study in breeding tree swallows

In this study, we quantified the average (population-level) response, amount of within-individual variation and repeatability of baseline concentrations of CORT (the primary avian GC) in wild breeding female tree swallows, as follows: (i) across breeding stages (from incubation to offspring provisioning); and (ii) in response to a feather-clipping manipulation during nestling provisioning that increases workload and decreases adult foraging profitability. As the feather-clipping manipulation can be viewed as a surrogate for environmental disturbance by creating an extended (multi-week) decline in access to food availability, it allowed us to assess whether birds would be likely to respond in individually specific ways to an environmental change. If baseline CORT concentrations represent a readily detectable indicator of environmental disturbance, we would predict that CORT concentrations would change at the population level, and across all individuals in the same manner (i.e. we predict that consistency repeatability will be high), in response to the clipping manipulation.

## Materials and methods

### Study species and sampling protocol

We monitored a nest-box breeding population of tree swallows from late April to early July 2011. Tree swallows are a member of a group of birds known as aerial insectivores, which are experiencing precipitous population declines in North America (Nebel *et al*., 2010). They readily nest in artificial boxes and are highly philopatric to their breeding grounds (Winkler *et al*., 2004). A total of 96 nest boxes were located across two sites in Haldimand County, Ontario, Canada located 4 km apart: Taquanyah Conservation Area (42°57′N, 79°54′W) and Ruthven Park National Historic Site (42°58′N, 79°52′W). Boxes were grouped within fallow fields near active agricultural fields, wetlands and riparian areas along the Grand River.

We monitored boxes every 2 days during the nest-building phase and daily after detection of the first egg to record the date of the first egg laid (lay date), egg mass, clutch size, hatch date, mass of chicks at days 6 and 12 post-hatching, and the number of offspring that successfully left the nest (fledging success). In addition, 10 days after clutch completion (late incubation) and 12 days after offspring hatch (peak nestling provisioning), we captured adult females at the next box to record mass and wing length and to obtain a blood sample (<150 μl) through puncture of the brachial vein. Females were provided with a federal numbered aluminum band (Canadian Wildlife Service Permit 10808). Blood samples were obtained within 2 min of covering the nest hole to ensure sampling of baseline concentrations of CORT (Romero and Reed, 2005) and between 08.00 and 12.00 h to control for diel variation in hormone concentrations (see Madliger *et al*. 2015). Samples were stored on ice for up to 5 h until centrifuged to separate plasma and stored at −80°C until assay. All animal handling and experimental methodology was approved by the Canadian Wildlife Service (Permit CA 0266) and the University of Windsor's Animal Care Committee (AUPP #10-10).

### Experimental manipulation

As tree swallows acquire all of their insect food resources for self-maintenance and offspring provisioning on the wing (Robertson *et al*., 1992), we used a feather-clipping manipulation that alters flight performance and foraging profitability to induce an extended increase in workload in females. Similar feather-clipping manipulations have been shown to result in a decreased ability to acquire food resources in this species (Winkler and Allen, 1995), and the manipulation leads to a decrease in the number of foraging bouts compared with control birds in our population (Madliger *et al*., 2015). Thus, feather clipping in this species provides a good means through which to mimic declines in food supply that would be expected to occur following environmental degradation. When females were captured for banding and blood sampling at day 10 of incubation (immediately before hatching), we clipped every other primary flight feather (four feathers on each wing) at the base of the wing with scissors (Winkler and Allen, 1995; Ardia and Clotfelter, 2007) on a subset of females (*n* = 33). Control females (*n* = 40) were handled identically, but their feathers were left intact. Control and manipulated females were matched spatially across sites and temporally by date over the season. Our previous work confirmed that control and feather-clipped females do not differ in baseline CORT concentrations prior to the manipulation, but clipped females have significantly higher baseline CORT than control birds at the mid-nestling provisioning period 2 weeks later (Madliger *et al*. 2015). Feathers remain clipped until natural moult occurs after breeding (Stutchbury and Rohwer, 1990); therefore, this manipulation alters female foraging ability for the entire period of nestling provisioning.

### Hormone analysis

We quantified baseline concentrations of CORT using a previously validated enzyme-linked immunoassay (EIA; Assay Designs, Ann Arbor, MI, USA; Love and Williams, 2008) optimized for tree swallows. Briefly, samples were run in triplicate at a 1:40 dilution with 1.5% steroid displacement buffer. Plates were run using a standard curve created by serially diluting a kit-provided corticosterone standard (from 20 000 to 15.63 pg/ml). Laying hen plasma was used as a control (Sigma-Aldrich, Oakville, Ontario, Canada). We read assay plates at 405 nm using a spectrophotometer plate reader. Intra-assay variation was 7.9%, and inter-assay variation was 11.2%. In cases where concentrations fell below the detectable limit of the assay (0.74 ng/ml), samples were assigned this detection limit (eight of 146 samples). We have previously used the hormonal data collected here to address separate research questions (Madliger *et al*. 2015; Madliger and Love, 2016).

### Quantifying habitat features

As nest boxes across both sites in our colony are surrounded by a variety of habitat types, including fallow fields, riparian areas associated with the Grand River, roadways, active agricultural fields, wetlands and forests, our goal was to complete a more detailed quantification of variation in surrounding habitat type rather than simply including ‘site’ as a covariate in subsequent analyses. We therefore characterized the surrounding habitat of each individual box to allow for its assignment to a habitat ‘cluster’. Specifically, we used a geographical information system (ArcGIS 10.1; Esri) and a 2010 orthorectified SWOOP (South Western Ontario Orthography Project) satellite image (20 cm resolution) to quantify the following habitat characteristics surrounding each next box: (i) distance to forest; (ii) distance to hedgerow; (iii) proportion of high-insect (i.e. food) land-use types within a 200 m radius; (iv) proportion of high-insect (i.e. food) land-use types within a 1 km radius; (v) distance to the Grand River; and (vi) distance to a roadway. We chose these variables based on tree swallow nest site preferences, requirements and potential disturbances (Table 1).

**Table 1:** Habitat variables quantified around each nest box and relevance of each feature to breeding tree swallows

Habitat variable	Relevance to breeding tree swallows	Reference(s)
Distance to forest	Nest predators, such as raccoons (*Procyon lotor*) and black rat snakes (*Elaphe obsoleta*), and the interspecific nest competitor house wrens (*Troglodytes aedon*), which destroy tree swallow eggs, are associated with wooded areas	(Weatherhead and Charland, 1985; Finch, 1990; Rendell and Robertson, 1990; Parren, 1991; Durner and Gates, 1993; Dijak and Thompson, 2000)
Distance to hedgerow	Interspecific nest competitors (house wrens) are associated with wooded areas	(Finch, 1990; Rendell and Robertson, 1990; Parren, 1991)
Proportion of high-insect land-use type (200 m radius)	Fallow fields, wetlands and cattle pastures (extensive land-use types) provide insect food resources. During nestling provisioning, tree swallows primarily forage within 200 m of their nest box	(Robertson *et al*., 1992; McCarty, 1995; McCarty and Winkler, 1999)
Proportion of high-insect land-use type (1 km radius)	Fallow fields, wetlands and cattle pastures (extensive land-use types) provide insect food resources. During incubation and nestling provisioning, tree swallows can travel longer distances to forage. One kilometre was chosen to quantify a landscape scale where the amount of extensive land use has been associated with differences in reproductive success	(Robertson *et al*., 1992; Ghilain and Bélisle, 2008)
Distance to Grand River	The Grand River represents a primary foraging location during periods of inclement weather	C.L.M., personal observation
Distance to roadway	Roadways represent a high-risk habitat feature to tree swallows (owing to potential mortality or injury), and many passerine species are negatively influenced by roads indirectly (e.g. noise)	(Ashley and Robinson, 1996; Reijnen and Foppen, 2006; Kociolek *et al*., 2011)

We performed a principal components analysis based on the correlation matrix of these six untransformed variables (James and McCulloch, 1990). Two principal components that explained 79% of the variance in the original variables were chosen based on examination of a scree plot (D'Agostino and Russell, 2005) and were subjected to varimax rotation (Abdi, 2004) to produce two factor scores for each box. Variables associated with food availability loaded heavily onto factor 1, whereas variables associated with nest disturbance loaded heavily onto factor 2 (Supplementary material Table 1). We subsequently performed a cluster analysis (James and McCulloch, 1990) using expectation maximization (normal mixtures) clustering (Nathiya *et al*., 2010) to create two categories (clusters) of boxes based on their factor scores. The final number of clusters was validated based on two characteristics obtained from a discriminant function analysis, with cluster identity as the dependent variable and the original habitat variables as independent variables (Leimeister, 2010), as follows: (i) a highly significant Wilks's λ (Wilks's λ = 0.046; *P* < 0.0001) indicating that >95% of the total variance in the discriminant scores was explained by differences between groups (clusters); and (ii) investigation of the number of errors the discriminant function analysis produced; two clusters produced the lowest number of classification errors (1%). The analysis grouped all of the boxes at Taquanyah Conservation Area with a grouping (subset) of boxes at Ruthven Park into one cluster, leaving the remaining boxes at Ruthven Park to compose the second cluster. Overall, this indicates that, on the basis of habitat variables known to be relevant to breeding tree swallows, clustering may better characterize the landscape characteristics birds were exposed to during our experiment than site alone. Specifically, the first cluster of boxes was characterized by lower nest disturbance, lower proportion of high-insect land-use types and close proximity to the Grand River (see Madliger and Love, 2016, Table 1 and Fig. 1). The second cluster was characterized by higher nest disturbance, greater potential availability of food resources and lower access to the Grand River (see Madliger and Love, 2016, Table 1 and Fig. 1). Therefore, we used habitat cluster rather than ‘site’ in all analyses to provide better control for the environmental landscape characteristics associated with each nest box.

### Statistical analysis

Statistical analyses were performed in JMP 12 (SAS Institute) and R 3.2.1 (R Development Core Team, 2015). We used four analyses to characterize population-level and within-individual changes in CORT (i.e. to determine which scenario in Fig. 1 best approximates our data) in control and feather-clipped birds separately. By separating analyses, we can distinguish between the patterns associated with natural conditions and those occurring in the face of a change in environmental quality as they represent two separate scenarios (responses) with different implications in the context of conservation monitoring. Baseline CORT values were logarithmically transformed before all analyses in order to achieve normality of residuals. We mean-centred continuous fixed effects in all analyses (i.e. we adjusted all fixed effects to a mean of zero by subtracting the average from each individual value). Mean-centring standardizes fixed effects, allowing the mean of the response variable to be interpreted as the mean phenotype in the average environment and an estimate of between-individual variance at the position in phenotypic space where fixed effects equal zero (Dingemanse and Dochtermann, 2013; Nussey *et al*. 2007). Data included here have been used in a previous publication (Madliger *et al*., 2015), in which we addressed separate research questions.

First, we tested for the equality of variances in baseline CORT between the incubation and nestling provisioning stages (control birds), and before and after the clipping manipulation (treatment birds), using a Bartlett test to determine whether the spread of baseline CORT values increased, decreased or remained the same over time. After logarithmic transformation, baseline CORT values were normally distributed at both reproductive stages in both clipped and control birds (Shapiro–Wilk tests: all *P* > 0.23). In addition to indicating whether the assumption of homogeneity of variances is met for subsequent analyses, this analysis is necessary to distinguish between scenarios c and d, or g and h, in Fig. 1.

Second, we determined whether baseline CORT changed from the incubation to the nestling provisioning stage (i.e. over a 2 week period) in control and clipped birds separately using a repeated-measures ANCOVA, with habitat cluster included as a random effect and laying date included as a fixed effect. This mimics a cross-sectional analysis and determines whether there is a difference in baseline CORT at the average (population) level between the incubation and nestling provisioning stage, or in response to the feather-clipping manipulation. Traditionally, cross-sectional analyses would sample random subsets of individuals at each time point; however, because our goal was to quantify the potential within-individual patterns that might be present, our analysis necessitated the use of repeated-measures data. We presented a similar analysis in a previous publication (Madliger and Love, 2015), but here we consider habitat type and reproductive investment and therefore re-present the data because they are integral to the interpretation of our subsequent analyses.

Third, we tested for differences in average baseline CORT concentrations between individuals (i.e. significant intercepts or ‘reaction norm elevation’, sensu Nussey *et al*., 2007). This analysis determines whether, on average over the two sampling times, individuals differ in their baseline CORT concentration. For example, the individuals in Fig. 1a and e would show significant between-individual variation in baseline CORT, whereas the individuals in Fig. 1b and f would not. We tested this specifically by comparing two hierarchical models with the same fixed-effect structure, but differing random-effects structure using a likelihood ratio test (LRT; Nussey *et al*., 2007; Montiglio *et al*., 2010; Kluen and Brommer, 2013). Likelihood ratio tests test for the significance of random effects by comparing the log-likelihoods of two nested models estimated with REML by using a χ^2^ distribution (Pinheiro and Bates, 2000). We constructed two models that both included baseline CORT as the dependent variable and habitat cluster (random) and reproductive stage, lay date, clutch size and brood size (fixed) as independent variables. In addition, one model included individual identity as a random effect to test for the significance of between-individual variance in baseline CORT concentrations. Likelihood ratio tests were completed using the lrtest function within the lmtest package in R (Hothorn *et al*., 2015).

Finally, we calculated the repeatability of baseline CORT in control and clipped groups separately. Repeatability is calculated as the variance between individuals divided by the total variance (the sum of between- and within-individual variance; Lessells and Boag, 1987). Most estimates of repeatability refer to ‘agreement repeatability’, where high estimates indicate low within-individual variability in absolute measures of a trait (Biro and Stamps, 2015). We also calculated ‘consistency repeatability’, which allows for high estimates of repeatability despite a change in a trait over time, as long as all individuals change in the same way (Nakagawa and Schielzeth, 2010; Biro and Stamps, 2015). As a result, consistency repeatability allows us to assess whether individuals show similar changes in GCs in response to a change in environmental quality. We calculated consistency repeatability by centring baseline CORT values on their mean at each measurement time (incubation and nestling provisioning; rather than using raw baseline CORT values, which leads to the calculation of agreement repeatability; as per Nakagawa and Schielzeth, 2010; Dingemanse and Dochtermann, 2013). We then used linear mixed-effects models controlling for habitat cluster, lay date, clutch size and brood size as fixed effects to determine repeatability in R 3.2.1 (R Development Core Team, 2015) using the package rptR (Nakagawa and Schielzeth, 2010). We included metrics of reproductive investment (clutch size and brood mass) to control for the possibility that high within-individual variation in baseline GCs over breeding, and in response to the clipping, could be related to reproductive workload (e.g. Bonier *et al*. 2011). To allow for subsequent logarithmic transformation of the CORT data, we added a constant to the mean-standardized values so that the lowest value was 1.00. We did not use a traditional random regression approach (e.g. Brommer *et al*., 2005; Nussey *et al*., 2007; Dingemanse and Dochtermann, 2013) to test for individually specific responses in baseline CORT (i.e. slope or ‘plasticity’) for two primary reasons: (i) sample size requirements for the determination of statistically significant individual plasticity are outside of those easily obtained in many wild populations (e.g. 200 observations; Martin *et al*., 2011), particularly for physiological data requiring blood sampling; and (ii) such approaches are better suited to experimental designs with more than two repeated measures per individual (Martin *et al*., 2011).

## Results

The overall variance in baseline CORT concentrations was equal at incubation and nestling provisioning in both control (Bartlett test, *F* = 0.87, d.f. = 1, *P* = 0.35) and clipped birds (Bartlett test: *F* = 1.54, d.f. = 1, *P* = 0.23).

**Figure 2:**
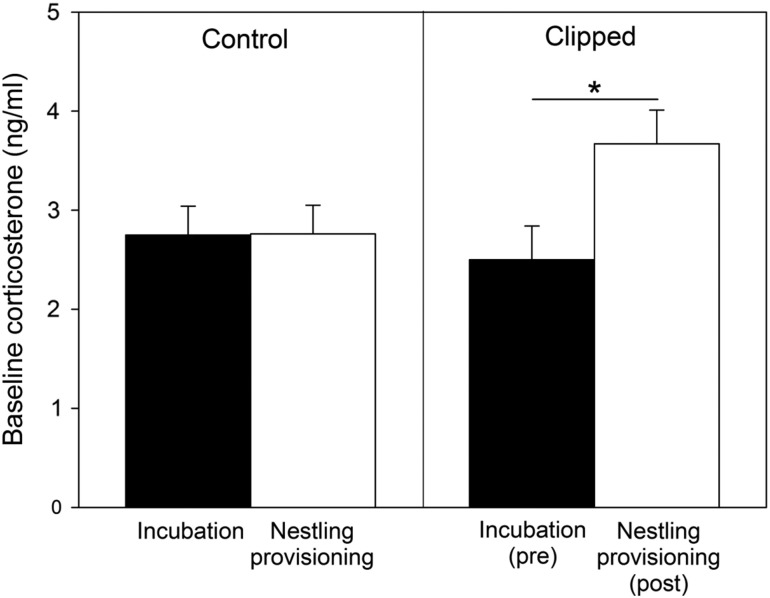
Differences in baseline corticosterone between the incubation and nestling provisioning stage in control (*n* = 40) and feather-clipped female tree swallows (*n* = 33). Values are shown as means ± SEM. Birds were assigned to a treatment group immediately after the incubation sample. *Statistically significant difference (*P* < 0.05).

There was no difference in average baseline CORT concentration between the incubation and nestling provisioning stage in control birds (repeated-measures ANCOVA, *F*_1,37_ = 0.30, *P* = 0.59; mean ± SE, incubation = 2.75 ± 0.29 ng/ml, nestling provisioning = 2.76 ± 0.29 ng/ml; Fig. 2). In the clipped group, baseline CORT at nestling provisioning (post-clipping) was significantly higher than during incubation (pre-clipping; repeated-measures ANCOVA, *F*_1,30_ = 9.41, *P* = 0.005; mean ± SE, incubation = 2.50 ± 0.34 ng/ml, nestling provisioning = 3.67 ± 0.34 ng/ml; Fig. 2), indicating that, on average, the clipping manipulation increased baseline CORT concentrations.

Control birds showed significant individual differences in baseline CORT (LRT, χ^2^ = 6.73, d.f. = 1, *P* = 0.009), indicating that birds differ in their average CORT concentrations (i.e. presence of between-individual variation in baseline CORT concentrations). In contrast, clipped birds did not show individual differences in baseline CORT (LRT, χ^2^ = 1.47, d.f. = 1, *P* = 0.23), indicating low between-individual variation.

**Figure 3:**
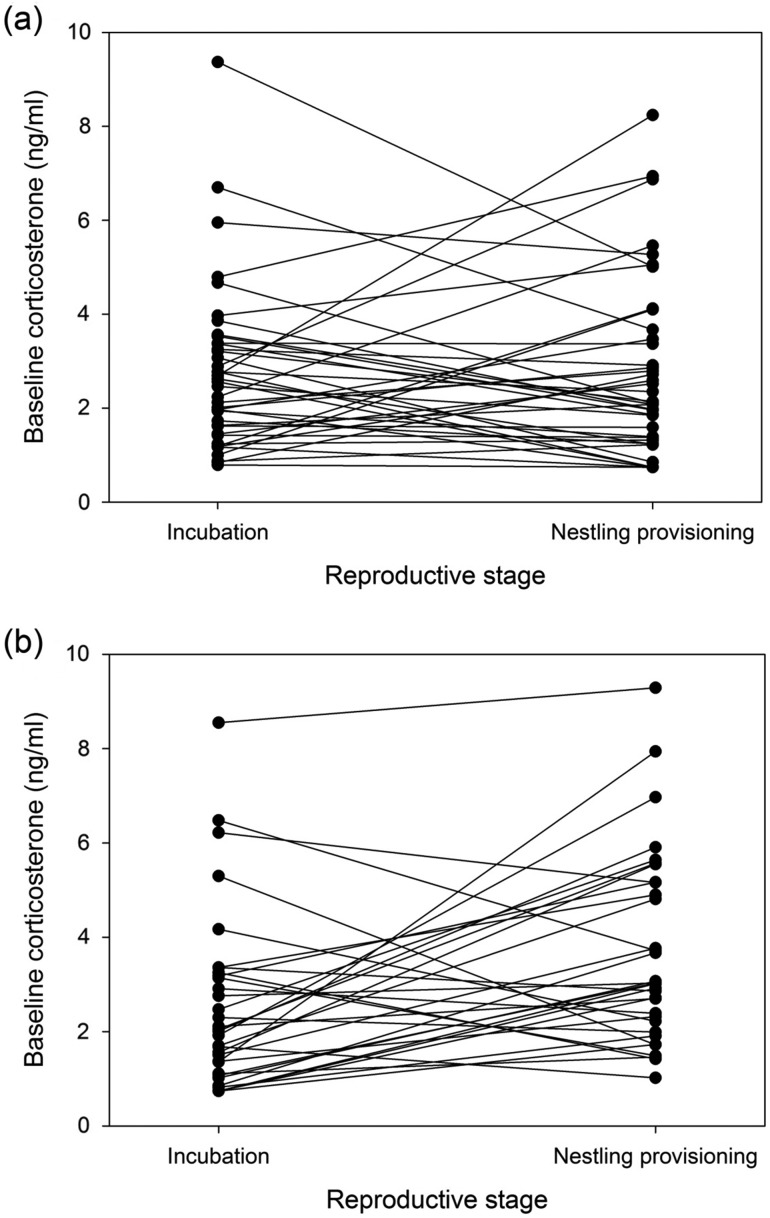
Individual changes in baseline corticosterone from the incubation to the nestling provisioning stage in control birds (*n* = 40; **a**) and feather-clipped birds (*n* = 33; **b**). Birds were assigned to a treatment group immediately after the incubation sample.

Baseline CORT concentrations were repeatable from the incubation to the nestling provisioning stage for control birds [consistency *r* = 0.42, SE = 0.14, confidence interval (CI) = 0.10–0.65, *P* = 0.006; Fig. 3]. Importantly, agreement repeatability estimates were equally high in control birds (*r* = 0.42, SE = 0.13, CI = 0.12–0.63, *P* = 0.006), indicating that this high repeatability was attributable to individuals maintaining the same absolute CORT values over time, as opposed to individuals changing similarly in a directional way across breeding. Baseline CORT concentrations for clipped birds were not repeatable (consistency *r* = 0.24, SE = 0.16, CI = 0–0.53, *P* = 0.10; Fig. 3; see Table 2 for associated within- and between-individual variance components). In the case of both control and clipped birds, repeatability estimates were similar regardless of whether lay date, reproductive investment and habitat type were included as covariates.

**Table 2:** Within-individual (residual) and between-individual (individual) variance components of mean-centred baseline corticosterone concentrations in breeding female tree swallows

Analysis	Parameter/variable		
**Control**		**Random-effects variance**	**SD**
	Individual	0.027	0.16
	Residual	0.036	0.19
		**Fixed-effects estimates**	**SE**
	Stage	−0.017	0.04
	Lay date	−0.001	0.005
	Clutch size	−0.008	0.04
	Brood size	0.0003	0.002
	Habitat type	−0.004	0.08
**Clipped**		**Random-effects variance**	**SD**
	Individual	0.012	0.11
	Residual	0.037	0.19
		**Fixed-effects estimates**	**SE**
	Stage	−0.018	0.05
	Lay date	−0.004	0.006
	Clutch size	0.045	0.05
	Brood size	−0.0009	0.001
	Habitat type	0.009	0.06

## Discussion

By quantifying the average change in baseline CORT, equality of variances, individual differences in average baseline CORT and repeatability, we were able to determine the population-level and within-individual patterns (Fig. 1) of baseline CORT that female tree swallows exhibit over the reproductive season and in response to a decline in foraging profitability. In natural conditions, neither the average nor the overall variance in baseline CORT concentrations differed between the incubation and nestling provisioning stage. We detected significant between-individual variance in baseline CORT concentrations, and values were repeatable within individuals. Overall, our repeated-measures data for control birds most closely approximates the pattern in Fig. 1e. These results are in line with previous work in tree swallows reporting consistency in baseline CORT concentrations over the breeding season when individuals are experiencing a non-manipulated environment (Ouyang *et al*., 2011a), and similar patterns over the breeding season have also been found in great tits (Ouyang *et al*. 2011a), male cane toads (*Rhinella marina*; Narayan *et al*., 2013) and female Fijian ground frogs (*Platymantis vitiana*; Narayan and Hero, 2013). It has been suggested that consistency in baseline GCs over the reproductive period may indicate that individuals make investment decisions and, in predictable conditions, maintain a constant investment level throughout the entire reproductive attempt (Ouyang *et al*., 2011a; Narayan *et al*., 2013). In addition, our results indicate that, in natural conditions, a single GC measure may be able to characterize an individual's baseline GC phenotype. This has important conservation implications, as it would suggest that researchers have a larger window over which GC measures can be taken and considered comparable. However, it is only through the additional investigation of such measures in relation to fitness that the full utility of baseline GC measures, and appropriate sampling regimes, can be ascertained.

In contrast to control birds, females that faced a decrease in foraging profitability (i.e. experienced an environmental change) had significantly higher average baseline CORT concentrations at the nestling provisioning stage (post-manipulation) in comparison to the incubation stage (pre-manipulation). As this manipulation has previously been shown to result in a decreased number of foraging trips in control birds (Winkler and Allen, 1995; Patterson *et al*., 2011; Madliger *et al*., 2015), it represents a biologically relevant proxy of a decrease in available food resources for females for both themselves and to provision their dependent offspring. As a result, a test of individual responses to this manipulation can provide insight into how females might respond to unexpected changes in the environment that manifest as decreases in food acquisition or other energetic constraints. We found evidence of individually specific responses in baseline CORT in response to the manipulation of foraging ability. Specifically, feather-clipped birds most closely approximated the pattern in Fig. 1b. The low repeatability estimate indicates that the amount of within-individual variation in baseline CORT was greater than the degree of between-individual variation; therefore, birds showed various types of baseline CORT responses to the feather-clipping treatment. A recent meta-analysis across taxa indicated that a common GC profile of chronic disturbance does not exist across species, with the authors concluding that it may be much more important to document the presence of any change, rather than a change in a specific direction (i.e. an increase; Dickens and Romero, 2013). Our results provide information on the potential for non-consensus in response at an even finer scale (within populations) and indicate that baseline GCs do not appear to change in a predictable way in response to a prolonged perturbation within a single population.

Low estimates of repeatability are in line with the labile role of baseline GCs in allowing individuals to respond to differing metabolic needs over time, and this flexibility has been considered adaptive (Bonier *et al*., 2009b). For example, the ability to modulate baseline GC concentrations has probably promoted range expansion in the invasive house sparrow (*Passer domesticus*) across Kenya (Martin and Liebl, 2014). More broadly, changes in baseline GCs may promote reallocation of resources during energetically demanding times of the life cycle (Love *et al*., 2004; Bonier *et al*., 2009a, 2009b; Ouyang *et al*., 2011b; Escribano-Avila *et al*., 2013). In our system, variation in the ability of males to compensate for decreased female provisioning rates (Madliger and Love, 2016) might have also contributed to the relatively low repeatability we observed in responses to the feather-clipping manipulation. This lack of repeatability also has implications for the application of baseline GCs as conservation biomarkers as it reveals that individuals can respond in individually specific ways to the same environmental perturbation. Importantly, our results do not differ when investment level, timing, female body mass and habitat type are considered, indicating that these additional contexts do not explain the variation in responses we observed.

In particular, the lack of consistency in response to environmental change that we observed highlights three possible complications. First, when the ability to obtain large, random samples is low, the presence of a high degree of within-individual variation could lead to false conclusions regarding the overall trend in the population. This is particularly relevant for conservation settings, particularly for physiological measurements where samples are often limited and invasiveness can be a concern. Second, if there are non-linear relationships between baseline GC concentrations and fitness, or if certain individuals that are the most important for population persistence are disproportionately affected by a disturbance, a cross-sectional approach could lead to under- or overestimation of the potential consequences of an environmental change. In both cases, these circumstances could cause misclassification of the level of conservation concern assigned to certain populations, leading to missed opportunities for mitigation or unnecessary effort. This is an important consideration to continue investigating as the tool is being developed for on-the-ground use in conservation settings. Finally, the presence of a large degree of within-individual variation might signal that individuals are responding in diverse ways to environmental alterations, potentially necessitating multiple measures of GCs over time in order to understand fully how responses might relate to fitness (an integral relationship to establish when using GCs as predictive biomarkers; Busch and Hayward, 2009; Madliger and Love, 2014). Given that a single measure may not characterize an individual's baseline GC phenotype during times of environmental change, it might be necessary to obtain multiple measures to predict subsequent fitness consequences.

It is possible that baseline GCs were changing based on the energetic demands imposed by inter- and intraspecific competition, food availability, paternal care, temperature or other weather conditions. However, our previous work did not find relationships between baseline GC concentrations in feather-clipped females and a number of environmental factors relevant to breeding tree swallows (food resources, inter- and intraspecific competition; Madliger and Love, 2015), and many of the other factors would be difficult to account for (e.g. parental care) or would lack relevance (e.g. labile/short-lived changes in GCs in response to temperature) in a conservation setting. We also eliminated or controlled for other contexts, such as broad age category, sex, life-history stage and timing of sampling, that could influence baseline GC concentrations. Consequently, our results indicate that single measures of baseline GCs might not be broadly representative of an individual's response to changing environmental conditions, and it might be much more important to assess how flexibility in hormone concentrations over time may be allowing individuals to cope with environmental and life-history demands (Bonier *et al*., 2009b, 2011; Ouyang *et al*., 2011b; Love *et al*., 2014). Overall, the observation of changing baseline GC concentrations at the population (average) level (based on single time point measurements from subsets of individuals) may not be adequate to draw conclusions about disturbance or health.

It is also possible that other perturbations in the environment could cause more consistent responses in baseline GCs or that other stages of the life cycle might be better suited to interpreting GCs as biomarkers. For example, the underlying demands associated with breeding (or other stages, such as migration) may impart difficulty in assessing baseline GCs as a biomarker of disturbance, whereas non-breeding seasons might show higher consistency. However, individuals still cope with alternative demands, habitats, timing and social interactions in the non-breeding season that can influence baseline GC concentrations (Marra and Holberton, 1998; Lindström *et al*., 2005; Garcia Pereira *et al*., 2006; Baker *et al*., 2013). It will therefore be important to investigate how individuals respond to perturbations of different intensities and durations across seasons, sexes and environments in order to ascertain fully the value of baseline GCs as conservation biomarkers. Our study was also completed during a single year in a short-lived species, making it important to investigate similar questions over longer temporal scales, or in years with harsher environmental conditions, for example. By examining the contexts that may drive high within-individual variation in baseline GCs across a variety of situations, managers could focus their efforts on segments of the population that are most impacted by a given disturbance (age class, sex, location, migratory route, etc.). In addition, we urge researchers to validate similar questions across different sampling media. For example, faecal GCs are appealing for conservation settings because of their low invasiveness and storage requirements, and such measures might be more easily validated in the context of individual variation because repeated measurements of faecal, rather than plasma samples, are more feasible for many species. However, it will nonetheless be important to tie samples to specific individuals and consider the extended time frame over which faeces integrate GC metabolites (Sheriff *et al*., 2011).

Although our study reveals information about intra-individual variation in baseline CORT in response to environmental change, we acknowledge that it involves limitations. The effects of our clipping manipulation coincided with a change in breeding stage (i.e. a transition from incubation to provisioning young). However, given that baseline CORT concentrations did not change in control birds across breeding stages, we are confident that the changes we observed in manipulated birds can be attributed to the constraints imposed by the clipping manipulation, rather than a natural change in workload over breeding. Furthermore, in terms of statistical analysis, we did not use a random sampling approach to investigate the difference in average CORT concentrations between incubation (pre-clipping) and post-clipping for two reasons. First, small sample size would affect our ability to draw strong conclusions if our data were partitioned into random samples at each breeding stage. Second, and most importantly, we aimed to take the most conservative approach to investigating underlying intra-individual variation. The possibility that the exact same data set could lead to alternative conclusions regarding how individuals might be responding to environmental change particularly illustrates the importance of examining data at multiple scales. Overall, our goal was to draw attention to a consideration that has been underappreciated in the context of using GCs as conservation biomarkers in hopes of spurring additional, directed research on within-individual variation.

We conclude that the use of baseline GCs may be limited in some wild systems or may require repeated measures and careful attention to context to determine fully how individuals are coping with extended disturbances in their environment. As a result, we encourage others to assess within-individual variation rather than relying on purely cross-sectional approaches before interpreting GC (and other hormonal) data (Williams, 2008). As information accumulates on the consequences of this type of variation for population persistence, we will be able to refine GC techniques to determine their relative role in the conservation toolbox.

## Supplementary Material

Supplementary Data
