# Supplementary material for: Employing individual measures of baseline glucocorticoids as population-level conservation biomarkers: considering within-individual variation in a breeding passerine
Source: Conserv Physiol. 2016 Oct 15;4(1):cow048. doi: 10.1093/conphys/cow048 (PMC5066389; doi:10.1093/conphys/cow048)
Supplement: Supplementary Data [file cow048_suppl_table_1_conphys_2016_046.docx]

**Supplementary Table 1** - Rotated factor loadings for habitat variables associated with tree swallow nest boxes.

| **Habitat variable** | **Factor 1** | **Factor 2** |
| --- | --- | --- |
| Distance to road | 0.14 | 0.86 |
| Distance to forest | 0.15 | -0.55 |
| Distance to hedgerow | 0.37 | 0.70 |
| Distance to Grand River | 0.58 | -0.67 |
| % high insect landuse (200 m) | 0.97 | 0.08 |
| % high insect landuse (1 km) | 0.88 | 0.02 |
|  |  |  |
| Proportion of variance | 0.40 | 0.39 |
